# Direct Cardiac Reprogramming in the Age of Computational Biology

**DOI:** 10.3390/jcdd11090273

**Published:** 2024-09-04

**Authors:** Rachelle Ambroise, Paige Takasugi, Jiandong Liu, Li Qian

**Affiliations:** 1 Department of Bioinformatics and Computational Biology, University of North Carolina, Chapel Hill, NC 27599, USA; 2 McAllister Heart Institute, University of North Carolina, Chapel Hill, NC 27599, USA; 3 Department of Pathology and Laboratory Medicine, University of North Carolina, Chapel Hill, NC 27599, USA

**Keywords:** direct cardiac reprogramming, computational biology, bioinformatics

## Abstract

Heart disease continues to be one of the most fatal conditions worldwide. This is in part due to the maladaptive remodeling process by which ischemic cardiac tissue is replaced with a fibrotic scar. Direct cardiac reprogramming presents a unique solution for restoring injured cardiac tissue through the direct conversion of fibroblasts into induced cardiomyocytes, bypassing the transition through a pluripotent state. Since its inception in 2010, direct cardiac reprogramming using the transcription factors Gata4, Mef2c, and Tbx5 has revolutionized the field of cardiac regenerative medicine. Just over a decade later, the field has rapidly evolved through the expansion of identified molecular and genetic factors that can be used to optimize reprogramming efficiency. The integration of computational tools into the study of direct cardiac reprogramming has been critical to this progress. Advancements in transcriptomics, epigenetics, proteomics, genome editing, and machine learning have not only enhanced our understanding of the underlying mechanisms driving this cell fate transition, but have also driven innovations that push direct cardiac reprogramming closer to clinical application. This review article explores how these computational advancements have impacted and continue to shape the field of direct cardiac reprogramming.

## 1. Introduction

### 1.1. The Heart’s Limited Regenerative Capacity

Since 1921, heart disease has been the leading cause of death in the U.S. [1]. Over a century later, heart disease continues to be the leading cause of death, not only in the U.S. but also globally, resulting in over 19 million reported deaths in 2020 and, more recently, over 20 million deaths in 2021 [2,3]. A significant proportion of these deaths are attributable specifically to ischemic heart disease, a condition in which the heart fails to receive adequate blood supply due to coronary artery disease or myocardial infarction (MI) [4].

It is estimated that more than a billion cardiomyocytes are lost after MI [5]. With there being only 3–4 billion cardiomyocytes in an average adult human heart [6], this substantial loss of contractile tissue can be devastating. It has been speculated that the human heart can only regenerate cardiomyocytes at a turnover rate of around 1% annually for adults aged 20, decreasing to about 0.3% by age 75 [7]. Due to the adult human heart’s limited regenerative capacity, dead cardiomyocytes in the infarcted region are replaced by fibroblasts, producing a rigid, fibrotic scar in a maladaptive process referred to as cardiac remodeling [8]. Since this scar tissue cannot contract, the remaining viable contractile tissue is placed under increased strain over time to continue satisfying the oxygen demands of the body, resulting in the progression to heart failure and, eventually, death.

Current treatments for heart disease are limited to symptom management through the use of medication to regulate factors such as blood pressure, fluid levels, and vessel constriction, and through the use of percutaneous coronary intervention (PCI) and stent placement to reestablish blood flow in previously blocked vessels [9,10]. While these treatments can prolong life expectancy and improve quality of life, they ultimately cannot reverse the damage incurred to the heart or avoid eventual heart failure and death. Several methods have been explored to address this issue. Heart transplants are a possible solution to extend the lifespan of heart failure patients; however, the demand for donor hearts far exceeds the supply [11]. Even after successful transplantation, patients can face severe challenges from post-operative complications such as organ rejection and immunosuppression. 

Stem cell therapies have presented another possible opportunity to address this issue through the reprogramming of somatic cells into induced pluripotent stem cells and later cardiomyocytes that can replace the diseased myocardium [12,13,14]. However, challenges in ensuring adequate engraftment, function, and overall survival of these stem cells have limited the clinical translation of this approach [15]. A less immunogenic alternative is stimulating the proliferation of pre-existing cardiomyocytes to compensate for the loss in contractile tissue. Numerous studies have investigated ways to prompt cardiomyocytes to re-enter the cell cycle and proliferate by overexpressing cell cycle activators [16,17,18,19,20], by expressing certain transcription factors [21,22,23,24,25], and by expressing microRNAs with established roles in regulating cell cycle entry [26,27,28]. However, this approach runs the risk of causing uncontrolled cancerous proliferation of existing cardiomyocytes [29]. Furthermore, while both stem cell-based and cardiomyocyte proliferation-based therapies have demonstrated success in increasing the number of viable cardiomyocytes in the heart, none of these methods directly address the scar tissue formed by fibroblasts in the regions of infarcted tissue. Evidence has shown that untreated scar tissue presents both mechanical and immunological barriers to successful clinical translation of these approaches by physically hindering the engraftment of stem cells and the migration of newly proliferated cardiomyocytes to the injury site, while also promoting an environment marked by acute inflammation that can further impede the success of these regenerative therapies [30,31,32,33]. In recent years, direct cell reprogramming—which is the process by which fully differentiated somatic cells are directly induced into a different cell type— has emerged as a promising alternative [34]. This process makes use of already residing cells, eliminates the need for an intermediate pluripotent state, and can be used to directly target fibroblasts, making it an attractive alternative to traditional transplantation, stem cell-based, and proliferative therapies. 

### 1.2. Origins of Direct Cardiac Reprogramming

In direct cardiac reprogramming, cardiac fibroblasts can be directly induced into cardiomyocyte-like cells through the administration of only three developmental transcription factors—Gata4, Mef2c, and Tbx5—collectively referred to as GMT [35,36,37,38]. This process was first achieved in vitro using murine cardiac fibroblasts [35]. Gata4, Mef2c, and Tbx5 were selected from a larger panel of 14 cardiomyocyte-specific and developmentally critical transcription factors and epigenetic remodeling factors. After testing different cocktails by serially removing individual transcription factors, it was found that infection with GMT was sufficient to induce direct transition from cardiac fibroblasts to induced cardiomyocyte-like cells (iCMs) [35]. This was evidenced by the activation of cardiac-enriched alpha-myosin heavy chain (*αMHC*)—a marker specific to cardiomyocytes— conjugated to GFP, by the acquisition of sarcomeric markers of differentiated mature cardiomyocytes—cardiac Troponin T (*cTnT*), and α-actinin— and by the induction of a cardiac gene expression profile in reprogrammed cells. These iCMs resembled endogenous cardiomyocytes not only in terms of their gene expression profiles but also in terms of their chromatin structures, sarcomeric structures, intracellular electrical signaling, calcium oscillations, and contractile activity [35].

However, at the inception of this technique, flow cytometry results indicated that the GMT cocktail could only successfully reprogram about 7% of fibroblasts, with only 1% achieving spontaneous beating activity [35]. Results from later studies further suggested that, while effective, GMT-mediated reprogramming is still quite inefficient. As Chen et al. reported, only 22% of infected fibroblasts in their study exhibited a voltage-dependent calcium current without a spontaneous action potential, indicating that the electrophysiological changes expected of successful reprogramming were incomplete [37]. They also found that GMT-reprogrammed fibroblasts had poor survival rates and minimal cardiac gene expression following transplantation into injured murine heart tissue [37].

To address these growing concerns, later studies, as outlined in Table 1, worked to identify additional factors that increased reprogramming efficiency while also expanding their focus to include in vivo models. In 2012, retroviral expression of GMT in mouse models following ischemic cardiac injury attenuated the expected decline in cardiac function and reduced overall fibrosis [39]. Another study affirmed through genetic lineage-tracing that delivery of GMT post-ischemic injury successfully reprogrammed cardiac non-myocytes—predominantly composed of fibroblasts— into iCMs [36]. While GMT-based in vivo cardiac reprogramming exhibited limited efficiency, similar to that observed from in vitro reprogramming (10–15%), in vivo reprogramming produced iCMs that more closely resembled endogenous cardiomyocytes, which Qian et al. attributed to factors within the native environment of the heart that enhanced this process [36]. Both studies also identified additional factors —Hand2 [39] and Thymosinβ4 [36]— for their potential to improve reprogramming efficiency and cardiac repair. Another study identified a combination of microRNAs independent of GMT that could achieve reprogramming [40]. Several variations of the GMT cocktail have since surfaced, with additional factors that have been reported to increase reprogramming efficiency [39,41,42,43,44,45].

One of the most significant strides in increasing reprogramming efficiency was made in 2015 with the development of a single polycistronic construct that expressed optimal ratios of *Mef2c*, *Gata4*, and *Tbx5* [46]. Wang et al. tested different combinations of polycistronic constructs expressing *Mef2c*, *Gata4*, and *Tbx5* and discovered that the stoichiometry of the three reprogramming factors greatly influenced the efficiency of direct cardiac reprogramming [46]. The construct that produced the most efficient in vitro and in vivo iCM reprogramming has a relatively high expression of *Mef2c*, with low expression of *Gata4* and *Tbx5* [46]. Compared to the previously used separate Gata4/Mef2c/Tbx5 delivery, this polycistronic construct —referred to as MGT— increased the percentage of *αMHC*-GFP+ cells by 3-fold (from 5% to 15%) and increased the percentage of *cTnT*+ cells by 5-fold (from 5% to around 25%), with the percentage of double-positive *αMHC*-GFP+ and *cTnT*+ cells increasing from less than 5% to around 10% [46]. The success of the polycistronic construct has resulted in MGT, which is currently the widely accepted basis of direct cardiac reprogramming.

**Table 1:** Summary of Reprogramming Methods.

Reprogramming Method	Type of Study	Origin Cell	Efficiency	Similarity to Primary CMs	Refs.
GMT	in vitro	mouse CFs, TTFs	6.5% (CFs), 2.5% (TTFs) *αMHC*-GFP+/ *cTnT*+	cardiac gene expression profiles, chromatin structures, sarcomeric structures, intracellular electrical signaling, calcium oscillations, contractile activity	[35]
GHMT	in vivo	mouse CFs, TTFs	9.2% (TTFs), 6.8% (CFs) *αMHC*-GFP+/ *cTnT*+	calcium transients and action potential similar to neonatal ventricular CMs	[39]
GMT, +Thymosinβ4	in vitro, in vivo	mouse CFs	12%	iCMs formed junctions with CMs; similar intracellular calcium release and cell shortening, marker expression (αActinin)	[36]
MGT + Sall4 + Myocd	in vitro	Mouse CFs (with MI model)	30% *cTnT*+/*cTnI*+ cells/field	beating	[47]
Cre-mediated MGTH	in vitro, in vivo	mouse CFs	4.8–5.2% *cTnT*+	sarcomeric structures, calcium oscillations, contraction	[48]
Ascl1 + Mef2c	in vitro	mouse CFs	9.45% (A+M), 14.0% (A+MGT)	mature iCM phenotype	[49]
miR combo (miR-1, miR-133, miR-208, miR-499)	in vitro	mouse CFs	4% *Actn2*+	sarcomere and electrophysiological properties of mature CM	[50,51]
miR-1, miR-133, miR-208, miR-499, ± JAK Inhibitor I Treatment	in vivo	mouse CFs	1.13–5.28% (*αMHC*-GFP+); with JAK Inhib—13.42–27.94% (*αMHC*-GFP+)	gene expression, sarcomere organization, calcium oscillations, mechanical contractions	[40]
MGT polycistronic	in vitro, in vivo	mouse CFs	9.23% *αMHC*-GFP+/*cTnT*+	Spontaneous beating	[46]
hMGT133	in vitro	human fibroblasts	30–40% *cTnT*+/α-Actinin+	CM molecular signature, calcium oscillations, contraction	[52]
hMGT133 + TBX20	in vitro	human fibroblasts	30.3% *αMHC*+; 23.8% α-Actinin+	beating, calcium oscillation, energy metabolism	[53]

CFs = cardiac fibroblasts, TTFs = tail tip fibroblasts, CM = cardiomyocyte, iCM = induced cardiomyocyte.

### 1.3. Bioinformatics and Research in the Modern Era

**Figure 1:**
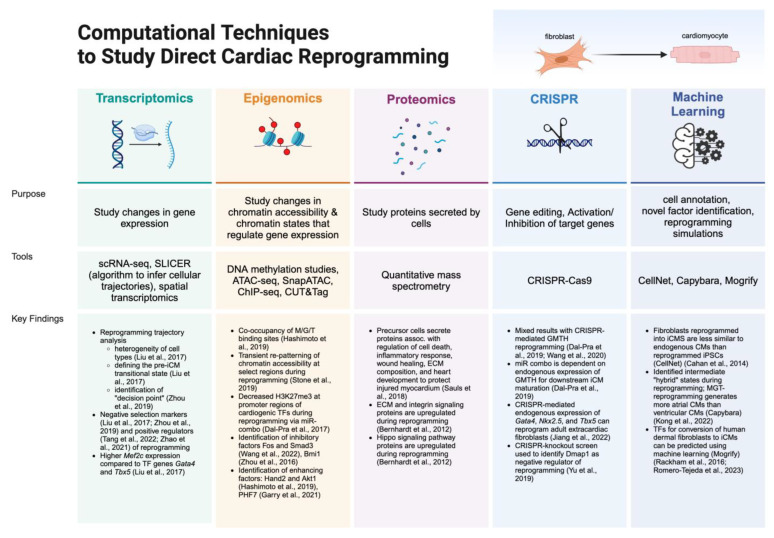
Overview of computational tools discussed in this review. Created with BioRender.com. References: Transcriptomics [47,52,53,54], Epigenomics [49,50,55,56,57,58], Proteomics [59,60], CRISPR [51,61,62,63], Machine Learning [64,65,66,67].

In the past decade, numerous studies have built on the foundation of the MGT cocktail, significantly advancing the field. The integration of bioinformatics —the application of computational tools, software, and statistical techniques to analyze complex biological data— has been integral to these advancements. In this review, we will explore key bioinformatics applications over the past decade that have propelled the field forward. As outlined in Figure 1, we will delve into the contributions of single-cell transcriptomic analyses to direct reprogramming and examine epigenomic analyses using chromatin immunoprecipitation and chromatin accessibility studies. Subsequently, we will review relevant proteomic analyses utilizing quantitative mass spectrometry and the insights they have provided on direct cardiac reprogramming. Following this, we will introduce emerging bioinformatics tools, such as CRISPR-Cas9 technology, spatial transcriptomics, and machine learning, which have yet to be extensively applied to the study of direct cardiac reprogramming. Collectively, these computational tools have been essential in elucidating the molecular mechanisms during the reprogramming process and in discovering additional factors and delivery methods to enhance reprogramming efficiency.

## 2. Transcriptomics in the Era of Single-Cell Analysis

One of the most important contributions of modern-day bioinformatics has been the ability to analyze snapshots of gene activity through transcriptomics analysis. Studying the changes in gene expression profiles has allowed us to not only gain insights into the regulatory networks driving direct cardiac reprogramming but has also helped to identify new target genes for optimizing this process. While both bulk and single-cell RNA sequencing (scRNA-seq) are important to the study of gene expression, this review focuses on the contributions of single-cell transcriptomics in the context of direct cardiac reprogramming.

Transcriptomics at the single-cell level has revolutionized the study of reprogramming. The first commercial single-cell RNA-seq platform became available in 2014 [68], with 10x Genomics’ Chromium for droplet-based high-throughput single-cell RNA sequencing only becoming available in 2016. Since then, the integration of single-cell transcriptomics into the study of direct cardiac reprogramming has been pivotal to advancing the field. By distinguishing transcriptomes at the level of individual cells, scientists have unveiled the heterogeneity of cell types at play throughout the reprogramming process. This review will detail some of the main findings from single-cell analyses in the context of murine fibroblast reprogramming, human fibroblast reprogramming, and reprogramming post-MI.

### 2.1. Insights from Single-Cell Analyses in Direct Cardiac Reprogramming of Murine Fibroblasts

As early as 2017, scRNA-seq analysis was used to study the reprogramming trajectory of murine fibroblasts induced by MGT and to uncover previously unknown intermediate cell subpopulations, gene pathways, and regulators involved in this process [54]. Using single-cell transcriptomics in conjunction with Selective Locally Linear Inference of Cellular Expression Relationships (SLICER) —an algorithm for inferring nonlinear cellular trajectories— the continuum of cell states during the reprogramming process was closely studied [69]. This allowed for the characterization of previously undefined intermediate states: the Fib (fibroblast), iFib (induced fibroblast), piCM (pre-induced cardiomyocyte), and iCM (induced cardiomyocyte) states [54]. Analysis of the gene networks specific to the iCM subpopulation compared against earlier cell subpopulations led to the discovery of novel negative selection markers *Cd200*, *Clca1*, *Tm4sf1*, and *Vcam*, as their expression levels were anti-correlated with the reprogramming process [54].

Along the reprogramming trajectory, *Mef2*c was, on average, more highly expressed than *Tbx5* and *Gata4* in the final iCM state, highlighting the uniquely important role that *Mef2c* expression plays in iCM induction [54]. Consistent with this finding, a later study used scRNA-seq data from GMT-based reprogramming to determine that while robust expression of *Mef2c* is required for direct cardiac reprogramming, high expression of the other two cocktail factors, *Gata4* and *Tbx5* were not as critical [56]. This was deduced from the observation that gene expression levels of *Gata4* and *Tbx5* varied widely among subclusters of cells along the trajectory from early to more distinct iCM reprogramming states, while all states required robust expression of *Mef2c*. 

An additional study of the pre-iCM cell subpopulation revealed that fibroblasts enter a transitional state where they are unstable and distinct from isolated starting fibroblasts as they express both CM (cardiomyocyte) and fibroblast markers [54]. This revealed a critical difference between induced pluripotent stem cell (iPSC) reprogramming and direct reprogramming: iPSC reprogramming requires an early downregulation of fibroblast markers to successfully progress, while direct cardiac reprogramming proceeds through an intermediate hybrid state before gradually suppressing fibroblast marker expression much later in the process as cells proceed toward the iCM state. 

### 2.2. Insights from Single-Cell Analyses in Direct Cardiac Reprogramming of Human Fibroblasts

Similar early applications of scRNA-seq analysis were later used to unveil the molecular framework behind direct reprogramming of human fibroblasts. One such application involved the integration of scRNA-seq data with SLICER to reconstruct the trajectory of human fibroblasts that were transduced with a human polycistronic *MEF2C*, *GATA4*, and *TBX5* construct along with microRNA-133 (hMGT133) toward becoming induced human induced cardiomyocytes (hiCM) [52]. RNA velocity analysis —which predicts if and how rapidly a given gene is being turned on or off— was applied to the reconstructed reprogramming trajectory. This analysis revealed that individual cells reach a “decision point” whereby they can respond to the hMGT133 cocktail and proceed toward an iCM fate referred to as the reprogramming pathway or regress toward a fibroblast fate in what is called the refractory route [52]. Further analysis of the single-cell transcriptomic data of the cells that undertook the refractory route was used to identify negative markers for reprogramming, including immune cytokine tumor necrosis factor alpha-induced protein 6 (*TNFAIP6*), metabolic enzyme aldo-keto reductase family 1 member C1 (*AKR1C1*), and fibroblast activation protein alpha (*FAP*) [52].

Other investigations of single-cell transcriptome data of hiCMs revealed additional factors that could improve reprogramming. For example, TBX20 was identified as a critical regulator of human cardiac reprogramming when single-cell transcriptome analysis of hiCMs induced by hMGT133 revealed that *TBX20* remained silent throughout the process, with hiCMs showing 100-fold lower expression of this gene compared to endogenous functional cardiomyocytes [53]. Validating this finding, overexpression of *TBX20* in conjunction with hMGT133 delivery drastically improved reprogramming efficiency and facilitated higher levels of spontaneous beating and actional potential generation in the reprogrammed hiCMs [53].

### 2.3. Application of Single-Cell Transcriptomics in Reprogramming after MI

Single-cell transcriptomics has been used to elucidate the underpinnings of cardiac reprogramming in the context of cardiac injury and infarction. In one such study, scRNA-seq data of embryonic cardiac cells were used to create a regulatory network of core transcription factors of cardiomyocyte identity. Screening TFs from this network led to the identification of Sall4 and Myocd as additional factors that, when added to the MGT cocktail, could significantly increase the in vitro reprogramming efficiency of cardiac fibroblasts isolated from adult mice with myocardial infarction (MICFs), promoting the subsequent spontaneous beating of cells [47]. A later study used a novel transgenic mouse model in which fibroblast lineage could be traced and reprogrammed via *Mef2c*, *Gata4*, *Tbx5*, and *Hand2* (MGTH) expression, which was induced in Cre-mediated druggable manner [48]. In this study, scRNA sequencing was used to determine the mechanism of cardiac repair by in vivo reprogramming in mice that underwent MI [48]. Analysis of the transcriptomic data from individual cells revealed seven different subpopulations of cardiac fibroblasts, two of which were enriched for genes related to activated fibroblasts. Furthermore, in vivo cardiac reprogramming showed a significant effect in reducing fibrosis post-MI, as overexpression of reprogramming factors converted profibrotic cardiac fibroblasts into a quiescent, inactive state [48]. In a third study, scRNA-seq data demonstrated that direct cardiac reprogramming post-MI could induce a prominent anti-inflammatory state [70]. While cardiac injury increases inflammatory pathway signaling, direct cardiac reprogramming appears to significantly suppress the inflammatory profiles of cardiac fibroblasts and reduce the relative ratios of pro-inflammatory signatures of cardiac macrophages post-MI [70].

Single-cell RNA-seq technology is one of the most impactful bioinformatics tools for the study of direct cardiac reprogramming. The ability to analyze changes in gene expression at single-cell resolution and at different time points during reprogramming has unveiled the complexity and heterogeneity of cell identities at play within direct cardiac reprogramming and has illuminated previously uncharacterized molecular mechanisms underlying this process in both native and injured cardiac tissue. In these ways, single-cell transcriptomic analyses have proven essential to inform current knowledge on the trajectories of individual cells during reprogramming and to identify critical factors that promote or inhibit successful transition to the iCM state. 

## 3. Advances in Epigenomics

The transition from fibroblasts to iCMs entails a substantial change in gene expression. Accomplishing such a drastic shift in the transcriptome necessitates overcoming epigenetic barriers for fibroblasts to adopt a cardiomyocyte-like chromatin pattern and cell identity. Analyzing the chromatin changes underlying this process is essential to improve our understanding of the molecular mechanisms driving direct cardiac reprogramming and to identify ways to optimize this transition. For these reasons, the application of epigenomics —or the study of the regulatory mechanisms and modifications that govern gene expression— to the study of direct cardiac reprogramming has proven to be of great importance. Two primary methods of analyzing such epigenetic changes are the Assay for Transposase-Accessible Chromatin (ATAC) sequencing and chromatin immunoprecipitation (ChIP) sequencing. 

ATAC-seq is used to study genome-wide chromatin accessibility patterns by using the hyperactive transposase Tn5 to preferentially fragment and tagment areas of open chromatin with sequencing adapters, creating a library that can be further analyzed using bioinformatics [71]. Alternatively, ChIP-seq is primarily used to identify the binding sites of DNA-associated proteins by crosslinking complexes of DNA to a protein of interest and extracting these protein-bound regions to construct a library for further analysis [72]. ChIP-seq was only first developed in 2007 [72], and the earliest form of ATAC-seq was created in 2013 [71], with both methods becoming commercially available and more widely adopted in subsequent years. As their names indicate, these are high-throughput sequencing methods that generate large datasets that require computational analysis. Parsing through these data has produced a wealth of knowledge on the inner workings of direct cardiac reprogramming. This review will highlight key findings from ChIP- and ATAC-seq studies with regard to insights into chromatin accessibility patterns, DNA-transcription factor interactions, and histone modifications that shape direct cardiac reprogramming.

### 3.1. Chromatin Accessibility Patterns in Direct Cardiac Reprogramming

Tools in epigenomics have allowed researchers to observe how changes in chromatin accessibility patterns facilitate changes in gene expression throughout the reprogramming trajectory. Liu et al. was the first group to characterize this repatterning in 2016 by tracking DNA methylation states of CpG sites within promoters of two representative cardiac genes: *Myh6* and *Nppa* [73]. In keeping with the goal of reprogramming to shift fibroblasts to iCMs, such sites were expected to be demethylated to allow for an increased CM-like gene expression profile. However, this study revealed that not every CpG site was equally demethylated during the early stages of iCM reprogramming, with certain CpG sites exhibiting greater demethylation and thus serving greater roles in regulating transcription during reprogramming. 

More recent studies have used ATAC-seq to analyze epigenomic repatterning during reprogramming. In one such study, ATAC-seq was performed on cells selected for the expression of *αMHC*-GFP [56]. These *αMHC*-GFP+ cells were collected at five different time points during reprogramming [56]. The data were analyzed for regions of accessible chromatin that differed from those observed in the starting fibroblast population [56]. Hierarchical clustering of the most differentially accessible regions revealed that most of these changes developed within three days of MGT induction and occurred distally from transcription start sites. Most regions associated with a stable gain in accessibility were highly correlated with the enrichment of sequence motifs specific to cardiovascular development. Regions that exhibited a sustained loss of accessibility were associated with inflammatory response mechanisms and monocytes, suggesting that effective reprogramming requires diminished inflammatory pathways. However, certain regions exhibited transient repatterning toward more open chromatin that quickly returned toward a more closed fibroblast-specific chromatin accessibility state at later time points, resulting in limited enrichment of transcription factor sequence motifs [56]. This may have prevented stable MGT binding, highlighting an epigenomic barrier to reprogramming efficiency. 

In another study, single-cell ATAC-seq data were integrated with scRNA-seq data using SnapATAC to delineate networks of transcription factors involved in the early shift of chromatin accessibility during cardiac reprogramming [74]. From this, a number of active transcription factors were identified for their time-specific roles in iCM conversion. For example, *Fos* —a gene encoding for a subunit of the heterodimeric transcription factor AP-1— was identified as a barrier for direct cardiac reprogramming that, when knocked down, improved reprogramming efficiency [74]. ATAC-seq data revealed this was because *Fos*-*AP1* motifs rapidly become inaccessible upon the induction of the iCM fate, resulting in its downregulation and, consequently, the suppression of fibroblast cell identity gene expression [74]. Interestingly, another transcription factor —*Smad3*— was found to play both inhibitory and supportive roles depending on the timing of its expression during reprogramming. Early in the process, Smad3 plays an inhibitory role by interacting with the heterodimeric transcription factor AP-1 to block the initiation of reprogramming. However, when active at intermediate stages, this same transcription factor facilitates reprogramming [74]. 

In the same study, scATAC-seq data were used to identify cis-regulatory regions gained by cardiac genes. Cardiac cis-regulatory regions were found to contain motifs of not only canonical MGT reprogramming factors, but also those of Tead family proteins, which are speculated to function as enhancers during reprogramming due to the enrichment of H3K27ac [74]. In these ways, ATAC-seq data have been used to reveal both the barriers and facilitators of direct cardiac reprogramming.

### 3.2. Applications of ChIP-Seq to Unveil Transcription Factor Interactions

One of the most advantageous results of applying ChIP-seq to the study of direct cardiac reprogramming has been the exposition of the genomic binding sites of reprogramming transcription factors. Hashimoto et al. were one of the first groups to use ChIP-seq in this way, analyzing both the genomic and epigenomic landscapes during direct cardiac reprogramming of mouse embryonic fibroblasts (MEFs) into induced cardiac-like myocytes (iCLMs) mediated by the GMT cocktail, a modified cocktail incorporating Hand2 (GHMT), and another modified cocktail incorporating an additional transcription factor Akt1 (AGHMT) [55]. Analysis of the binding sites for reprogramming transcription factors (TFs) Gata4, Hand2, Mef2c, and Tbx5 2 days after GMT-mediated reprogramming revealed considerable co-occupancy of reprogramming TFs, where the percentage of co-occupied peaks (or peaks occupied by at least two reprogramming TFs) increased with the addition of Hand2 (GHMT) and Aktl1 (AGHMT) [55]. This led to the discovery that reprogramming TFs are synchronously recruited to genomic sites to drive reprogramming and that Hand2 and Akt1 can enhance reprogramming by increasing the recruitment and subsequent co-occupancy of these TFs to sites that drive cardiac gene expression [55]. 

Hashimoto et al. also used the data from this ChIP-seq analysis to construct a gene regulatory network (GRN) of reprograming factors in day 2 AGHMT iCLMs [55]. By annotating all TF peaks in day 2 AGHMT iCLMs, comparing all upregulated genes in day 2 AGHMT iCLMs against those of mock-infected MEFs, and connecting each upregulated gene with the reprogramming TF responsible for driving its expression based on the nearest TF peaks, they were able to construct a GRN of the reprogramming factors with their potential target genes during reprogramming [55]. In silico analysis using the Protein Analysis Through Evolutionary Relationships (PANTHER) classification system [75]—a database containing gene ontology (GO) analysis— further revealed that the upregulated genes in this network were highly enriched for GO terms related to “muscle contraction, metabolism, cell-cell interaction, and ECM”, which is consistent with the transition of fibroblasts into cells of contracting muscle with increased metabolic demands that occurs during direct cardiac reprograming [55]. Downregulated genes in the GRN showed enrichment in terms associated with “cell cycle, ECM, and inflammation pathways”, all of which have been reported to enhance reprogramming when suppressed [55]. In these ways, ChIP-seq analysis has been an integral tool in uncovering the ways in which reprogramming TFs mechanistically drive the changes in gene expression observed during the reprogramming process. 

ChIP-seq has also been used to study epigenomic repatterning during reprogramming, whereby certain regions of chromatin become more or less accessible for transcription factor binding. As Stone et al. observed, ChIP-seq analysis revealed statistically significant enrichment of MGT binding in regions that gained accessibility during reprogramming [56]. There is additionally significant enrichment of motifs specific for several non-cocktail reprogramming transcription factors from families that include bZIP, Homeobox, and Forkhead proteins. This indicates that administration of MGT induces changes in chromatin accessibility that permit combinatorial binding of factors beyond MGT alone to facilitate reprogramming [56]. However, these changes are attributable only to Mef2c and Tbx5, as Gata4 was not observed to independently cause any stable increase in chromatin accessibility. Mef2c has been further highlighted for its uniquely important role in driving direct cardiac reprogramming, as ChIP-seq data revealed that histone marks associated with active enhancer elements were most dominantly associated with Mef2c binding sites compared to the binding sites of other reprogramming TFs [55]. The results of these studies suggest that while Mef2c and Tbx5 are critical drivers of epigenomic remodeling, Mef2c is also a critical transcriptional activator of reprogramming [55], whereas Gata4 likely plays a more downstream role in the reprogramming process [56]. 

Additional chromatin immunoprecipitation studies have helped clarify the function of Gata4 in reprogramming. In a 2017 study, it was observed that MGT administration to rat cardiac fibroblasts in vitro significantly downregulated the expression of Snail, *Col1a1*, fibronectin, and other profibrotic factors [76]. ChIP-qPCR identified Gata4 binding sites in the Snail promoter, revealing that Gata4 was responsible for the downregulation of Snail. This finding was further validated in a rat coronary ligation model, in which only Gata4 administration out of all the pioneer reprogramming factors was found to independently improve post-infarct ventricular function and reduce fibrosis. In a more recent study, Cleavage Under Targets and Tagmentation (CUT and Tag) analysis —a newer alternative method to profile the interactions between DNA and proteins— identified Gata4 as a critical target of the immune system’s response to resist direct cardiac reprogramming [77]. Wang et al. observed that cardiac fibroblasts that were transplanted into infarct regions of MI mouse models resisted reprogramming due to an upregulation of IFN response genes, such as *STAT1* [77]. CUT and Tag analyses revealed that phosphorylated STAT1 interacts with Gata4 in a way that inhibits Gata4 from binding to cardiac genes during reprogramming [77]. 

### 3.3. Histone Modifications in Direct Cardiac Reprogramming

Histone modifications are also important in dictating chromatin accessibility patterns. Along with DNA demethylation studies, Liu et al. used ChIP-seq to characterize the repatterning of chromatin during direct cardiac reprogramming [73]. This study revealed that histone marker deposition coincided with the early and rapid activation of cardiomyocyte-specific genes and progressive attenuation of fibroblast-specific genes. Specifically, H3K27me3 deposition, a marker of gene silencing, was reduced, and H3K4me3 deposition, a marker of gene activation, was increased at cardiac promoters as early as day 3 of reprogramming [73]. In contrast, H3K27me3 deposition at fibroblast-specific loci increased much later, around day 10, with H3K4me3 marks progressively decreasing [73]. These time-specific changes in histone marker deposition and removal shed light on the transcriptional shifts observed throughout the reprogramming trajectory. 

Other studies have identified potential non-cocktail regulators of direct cardiac reprogramming by analyzing the mappings of repressive and active histone marks. In 2016, ChIP-seq data of H3K4me3 and H3K27me3 genome mappings were analyzed in a reprogramming model that used cells depleted of Bmi1 –an epigenetic regulator known to mediate monoubiquitination of histone H2A to repress gene expression [57]. Knockdown of *Bmi1* led to an increase in H3K4me3 deposition at cardiogenic loci, indicating that Bmi1 plays an antagonistic role in direct cardiac reprogramming by reducing chromatin accessibility of CM-specific genes [57]. Another study in 2021 established that the histone reader PHF7 is a potent activator of direct cardiac reprogramming [58]. H3K27ac ChIP-seq analyses revealed that PHF7 localizes to cardiac super-enhancer regions in fibroblasts through its cooperation with the SW1/SNF chromatin remodeling complex, inducing increased chromatin accessibility for transcription factor binding [58]. Similarly, Dal-Pra et al. used ChIP-qPCR to investigate the mechanism of reprogramming mediated by a combination of microRNAs (miR-1, miR-133, miR-208, an miR-499), referred to as the “miR combo”. Their study showed decreased H3K27me3 deposition at promoter regions of cardiac transcription factors when treated with the miR combo, indicating that the miR combo facilitates fibroblast transition to iCMs by removing repressive histone marks [50]. In a more recent study, ChIP-seq data were used to validate the mechanism by which a neuronal transcription factor —Ascl1— could be used to enhance direct cardiac reprogramming [49]. It was determined that Mef2c induced a shift in the binding pattern of Ascl1, such that this neuronal TF binds to more cardiogenic sites [49]. In these ways, histone marker ChIP data have successfully been used to illuminate not only the mechanisms by which epigenomic remodeling occurs during reprogramming, but also to identify important non-cocktail repressive and activating factors that can be further studied to optimize this process. 

## 4. Advances in Proteomics

Mass spectrometry, which uses the mass-to-charge ratio of ions in a sample to analyze proteins, is one of the primary methodologies in proteomics. With the large-scale data produced from proteome studies, bioinformatics tools and software have become integral to analyzing this data. Many of the computational tools commonly used today, such as Proteome Discoverer, Mascot, MaxQuant, PEAKS, and SpectroNaut, were first developed between 2007 and 2012 and have since undergone numerous updates to improve their protein quantitation, characterization, and identification methods [60,78,79,80]. The use of proteomics —or the analysis of cellular states at the protein level— has contributed significant findings to our current understanding of the changes that occur during direct reprogramming. This review specifically explores how the use of quantitative mass spectrometry has afforded a greater understanding of direct cardiac reprogramming through the investigation of the proteins secreted during this process. 

### Findings from Mass Spectrometry

In 2018, a quantitative mass spectrometry (QMS)-based proteomics approach was used to analyze changes in protein abundance during the initial phases of iCM reprogramming [59]. It has previously been demonstrated that transduced fibroblasts undergo drastic repatterning of histone marks 3 days after MGT-mediated direct reprogramming, and this was correlated with both early activation of cardiac genes as well as progressive suppression of fibroblast genes [73]. To investigate whether these changes extend beyond the transcriptome, Sauls et al. applied QMS and performed gene set enrichment analysis of quantified proteins. Using the STRING database, they were able to investigate protein-signaling networks and identify time-specific changes in protein abundance that coincided with the transcriptomic changes observed earlier. For example, extracellular matrix (ECM) protein and integrin signaling protein abundances were the most significantly upregulated protein classes at both 2- and 3-days post-transduction, with Agrin —an ECM protein that inhibits Hippo pathway signaling and has been established to stimulate cardiac repair and proliferation— being the single most upregulated of all ECM proteins [59]. These findings highlighted the potential uses of inhibiting Hippo pathway signaling and driving integrin signaling to optimize conditions for the growth and proliferation of iCMs in direct cardiac reprogramming. 

Interestingly, however, ECM proteins were less strongly upregulated at 3 days post-transduction than at 2 days post-transduction, consistent with previous studies that showed a gradual suppression of fibroblast-specific genes. Other protein classes displayed more nuanced changes. For example, translation factor proteins were downregulated at 2 days post-transduction but not at 3 days, which they posited reflected an early initial need for accelerated protein synthesis as fibroblasts adopt the structural components of cardiomyocytes, followed by a decrease in protein synthesis by day 3 as energy is depleted from the cells [59]. Similarly, chromatin-binding proteins displayed downregulation in abundance at day 3, but not at day 2 post-transduction, suggesting that major chromatin remodeling events for reprogramming may have already been completed in the first 3 days [59].

A more recent study in 2023 used mass spectrometry to study the secretome of proteins produced by induced cardiomyocyte cell precursors (iCMPs) [81]. Studies have shown that transplantation of this intermediate cell type into infarcted mouse heart tissue reduces fibrotic scarring and preserves ventricular function. For this reason, proteomics was used to investigate whether these cardioprotective features could be attributed to specific factors secreted by iCMPs. Mass spectrometry data revealed that these precursor cells secreted proteins specific to the regulation of cell death, extracellular matrix composition, and heart development, among other processes [81]. Galectin-3 —a protein reported to reduce infarct size, promote wound healing, increase ventricular remodeling, support macrophage infiltration, and sustain heart function when present after injury— and S100-A10 —a protein associated with macrophage invasion and migration— were among the proteins secreted exclusively by iCMPs [81]. These findings substantiate the clinically relevant role of cardiac fibroblasts in secreting proteins that drive wound healing, inflammatory responses, and heart development processes to protect injured myocardium. 

## 5. Upcoming Bioinformatics Applications in Direct Cardiac Reprogramming

The integration of bioinformatics into the study of direct reprogramming is an ongoing development. A few burgeoning examples in recent years include the use of CRISPR-Cas9, spatial transcriptomics, and machine learning-based analyses. This review will detail any recent applications of these technologies in the study of direct cardiac reprogramming and explore their potential for future applications. 

### 5.1. Developments from CRISPR-Activation and -Inhibition Studies in Cardiac Reprogramming

CRISPR, short for clustered, regularly interspaced short palindromic repeats, has been a widely used tool in gene editing studies since its discovery over a decade ago [82]. This system makes use of a catalytically dead Cas9 (dCas9) that, when customized with single guide RNAs (sgRNAs), can regulate gene expression in a targeted manner. In the context of direct cardiac reprogramming, CRISPR presents itself as a powerful alternative to conventional reprogramming methods that overexpress exogenous reprogramming factors. In contrast, CRISPR mainly targets endogenous gene expression, as directed by sgRNAs. This presents a considerable difference in overall expression levels, as traditional reprogramming methods of exogenous GMT/GMTH overexpression increase the expression of these factors by over 10,000-fold, whereas miR combo-mediated reprogramming only increases endogenous GMTH expression by 1.5–5 fold [51]. Interestingly, despite the considerably smaller change in overall GMTH expression compared to traditional methods, the miR combo still induces reprogramming [51]. Studies employing this use of CRISPR have increased our understanding of the role of both endogenous and exogenous transcription factors in reprogramming [51,62].

The applications of CRISPR have shown mixed results. For example, Dal-Pra et al. employed CRISPR to study the role of endogenous reprogramming factors *Gata4*, *Mef2c*, *Tbx5*, and *Hand2* in the context of both GMTH- and miR combo-mediated reprogramming [51]. When Dal-Pra et al. used a dCas9 fused to transcriptional activator VPR to induce the expression of endogenous factors GMT, this CRISPR-mediated approach failed to reprogram fibroblasts into cardiomyocyte-like cells, despite inducing GMT expression at levels comparable to those observed by the miR combo. In a follow-up experiment using CRISPR-mediated inhibition, the miR combo, while successfully inducing reprogramming of fibroblasts, could not facilitate cardiomyocyte maturation when GMTH expression was inhibited [51]. This highlights the necessity of the miR combo to induce the expression of GMTH and the overall importance of high levels of GMTH for cardiomyocyte maturation.

Other studies, however, were able to derive success from CRISPR-mediated reprogramming. Jiang et al. used a CRISPR activation system to induce endogenous expression of factors *Gata4*, *Nkx2.5*, and *Tbx5* in adult extracardiac fibroblasts [62]. This resulted in successful reprogramming into cardiovascular progenitor cells (CPCs), which could even give rise to cardiovascular cells and restore contractile function when engrafted into infarcted heart regions [62]. This approach still has limitations in that the reprogrammed CPCs predominantly develop into vascular smooth muscle cells and endothelial cells (~24% and ~39%, respectively), with cardiomyocytes constituting a minority (~36%) [62]. Nonetheless, the success of this approach highlights a different set of reprogramming factors —GNT— that can be used to endogenously drive direct cardiac reprogramming. In another study, CRISPR-Cas9 was used to activate endogenous cardiac factors *GATA4*, *HAND2*, *MEF2C*, and *TBX5* in human fibroblasts [61]. This method of lineage reprogramming was able to successfully reprogram human dermal fibroblasts into induced cardiac progenitor cells (iCPCs) that can differentiate into three cardiac lineage cells: cardiomyocytes, smooth muscle cells, and endothelial cells [61]. 

CRISPR technology has also been used to optimize reprogramming through knockout screens that enable the subsequent identification of novel factors for improved cocktails. In 2019, Yu et al. used a CRISPR-Cas9 knockout model to explore the molecular mechanisms underlying reprogramming with a specific chemical cocktail [63]. Notably, this cocktail deviates from the canonical reprogramming MGT cocktail, as its main constituents are small molecule TGFβ-pathway inhibitors that are used to upregulate the necessary lineage-specific changes in gene expression to reprogram fibroblasts into progenitor cells [63]. The results of this study were later used to identify the factors and pathways that regulate this process. To do this, researchers used next-generation sequencing to examine the DNA of cells with high levels of *Nkx2-5*, a marker for cardiac cells, from their CRISPR-edited library. They then analyzed the data using the MAGeCK tool, which helps identify which gRNAs were significantly enriched, pointing to important genes involved in the process [63]. The results of the knockout screen revealed that among the top hits, guides targeting and inhibiting DNA methyltransferase 1 associated protein 1 (Dmap1) most consistently increased *Nkx2-5* expression. This suggests that Dmap1 acts as a negative regulator of direct cardiac reprogramming [63]. Further validation studies confirmed this, showing that the loss of Dmap1 led to a 50% reduction in *Nkx2-5* promoter methylation, which increased chromatin accessibility and likely facilitated *Nkx2-5* expression [63].

### 5.2. Future Applications of Spatial Transcriptomics to Study Direct Cardiac Reprogramming

As discussed in this review, transcriptomic analyses have been integral to the study of direct cardiac reprogramming, unveiling the changes in gene expression that must take place as cells transition from fibroblasts to cardiomyocyte-like cells. Single-cell RNA sequencing has enabled closer investigation of the different cardiac cell types at play during the reprogramming process. Nevertheless, these analyses, when applied in vivo, come with the caveat of losing all the organizational and positional information of these different cell types. 

Fortunately, in 2016, Ståhl et al. introduced spatial transcriptomics —a novel method for profiling quantitative changes in gene expression in a way that also maps the spatial organization of these changes within intact tissue [83]. This method makes use of unique positional barcodes that allow transcriptomic data captured from gene-specific probes to be mapped back to their physical origins within the tissue. While this tool is yet to be applied to direct cardiac reprogramming, studies have used spatial transcriptomics to better understand the organizational structure of the developing heart [84,85,86] and the changes in expression that occur in the context of cardiac disease [87,88,89].

One current limitation of applying spatial transcriptomics (ST) to the study of direct cardiac reprogramming is the need for careful spot deconvolution when using sequenced-based assays such as the Visium ST platform. The spatial resolution of sequence-based spatial transcriptomics approaches has drastically improved from 100 μm [83] to 55 μm, capturing 1–10 cells in each spot and eventually down to a resolution of 2 μm [90]. Nonetheless, proper deconvolution and segmentation methods are essential to ensure that heterogeneous and sometimes transient cell types at play during reprogramming can be accurately distinguished. Image-based spatial transcriptomics approaches, such as Xenium [91], have reported better success in capturing data at the subcellular level; however, these image-based approaches are far more limited in the number of RNA targets they allow compared to sequence-based approaches. These differences are important to consider when planning to integrate spatial transcriptomics into the study of direct cardiac reprogramming. 

Nonetheless, future studies on the spatial distribution of gene expression changes during direct cardiac reprogramming can provide valuable insights into how this process is directly affected by the native microenvironment. Such data would be clinically relevant to understanding, for example, how the spatial organization of the heart influences cellular responses to reprogramming and how reprogramming cocktails can be improved to selectively target infarct regions.

### 5.3. Potential Developments from Machine Learning in Cardiac Reprogramming

Machine learning —a branch of artificial intelligence in which computer systems can be trained to process and model data— is a recent and promising development in bioinformatics that can confer numerous advantages in the study of direct cardiac reprogramming. Isolating primary fibroblasts and culturing them for a sufficient time can be a taxing process. Computational modeling can be used to circumvent this issue, producing timely results for further experimental validation. Such advances can be critical for progressing the field of direct cardiac reprogramming. The following sections will explore current machine learning algorithms focused on cell identity annotation and cell reprogramming modeling that can potentially be applied to study direct cardiac reprogramming.

#### 5.3.1. Cell Identity Annotation Algorithms

Given the heterogeneous nature of cells, as they progress through transdifferentiation, a critical concern in direct cardiac reprogramming is properly annotating and identifying discrete and intermediary cell types. As previously discussed, the rise of bioinformatics has created large repositories of data for computational analysis to address this question. In 2014, Cahan et al. developed CellNet —a network biology platform to quantify how closely reprogrammed cell populations resemble their target cell type [64]. This platform was developed by reconstructing gene regulatory networks using around 3500 publicly available gene expression profiles from diverse cell types in both human and mouse tissues to train a Random Forest classifier. Findings from CellNet indicated that while iCMs from direct reprogramming via ectopic expression of *Gata4*, *Mef2c*, and *Tbx5* do resemble their endogenous CM counterparts, they are less similar to endogenous CMs than induced pluripotent stem cells (iPSCs) that are converted to iCMs [64]. This observation was attributed to the inadequate silencing of gene expression programs from the starting fibroblast population, thus highlighting a key area for improvement and future troubleshooting in direct cardiac reprogramming. 

Several computation-based algorithms have since been developed to annotate cell identity using scRNA-seq data, scATAC-seq data, or the integration of these data [92,93,94,95]. More recently, Capybara —a computational tool for classifying discrete cell identities and intermediate “hybrid” states—was developed [65]. This method assigns continuous identity scores to each cell against exhaustive public cell types using quadratic programming to capture the gradual transition in cell identity that occurs during reprogramming. Kong et al. applied Capybara to MGT-mediated direct cardiac reprogramming of cardiac fibroblasts to CMs and found that atrial CMs are generated in larger quantities than ventricular CMs [65]. This finding was used to support modified protocols that inhibit TGFβ-signaling with Wnt activation to optimize reprogramming and increase the relative yield of ventricular CMs [65].

#### 5.3.2. Computational Modeling and Reprogramming Factor Prediction

Compared to purely experimental approaches, machine learning can be used to screen for novel reprogramming factors and model reprogramming in silico in a time- and cost-efficient manner. One of the earliest examples of this is Mogrify —a platform that leverages transcriptomic data with regulatory network information to predict reprogramming factors necessary for specific cell conversions [66]. When applied to the conversion of human dermal fibroblasts into cardiomyocytes, Mogrify was able to predict four out of the five major transcription factors used in this conversion (GATA4, TBX4, HAND2, and NKX2.5). 

While MGT-based reprogramming has had wide success in studies involving mouse fibroblasts, this same cocktail has not been as efficacious when applied to human fibroblasts. To address this, Mogrify was able to successfully predict and identify novel transcription factor candidates to induce human direct cardiac reprogramming [67]. The authors then developed a high-throughput screening process using lentiviral transduction and a reporter system to screen all potential combinations of transcription factors. Ultimately, a combination of the factors identified by Mogrify —*MYOCD*, *SMAD6*, and *TBX20* (MST)— was found to successfully drive human direct cardiac reprogramming, with overexpression of MST consistently producing 40% of *TNNT2*+ cells over the course of 25 days [67]. While such computational reprogramming models will always benefit from additional experimental validation, the early success of Mogrify proves that machine learning can quickly shorten the time for factor discovery by modeling reprogramming in silico. 

Machine learning has already begun to inform studies on direct cardiac reprogramming. In 2019, Stone et al. developed a computational framework to model gene expression changes as a function of transcription factor-binding motifs in dynamic regions of open chromatin [56]. This model was used to identify new candidate factors during the first two days of GMT-based reprogramming. shRNA knockdown validated these predictions, showing a significant reduction in reprogramming efficiency when targeted toward predicted inhibitory factors *Sp1*, *Foxo1*, *Tcfp2l1*, *Tgif1*, and *Foxp1*, and increased reprogramming efficiency when targeted toward predicted facultative factors *Hif1a*, *Prdm1*, and *Smad3* [56].

Similar computational modeling methods have been developed. While some platforms do not have documented applications specific to direct cardiac reprogramming, their application to other reprogramming studies nonetheless serves as a proof of concept for future applications to direct cardiac reprogramming. For example, the single-cell Reprogramming Model Through cis-regulatory Elements (scREMOTE) is a platform that integrates both scRNA-seq and scATAC-seq data to calculate the regulatory potential for each given transcription factor [96]. These regulatory potentials are then used to build a regression model based on gene expression to estimate the effect of transcription factor perturbations on reprogramming [96]. 

Another platform called Reprogram-seq was developed to experimentally screen thousands of transcription factor combinations for reprogramming performance [97]. This method uses organ-specific cell atlas data with single-cell perturbations to predict the effects of different transcription factor cocktails. To screen for transcription factors that can convert fibroblasts to epicardial-like cells, Reprogram-seq was trained on single-cell transcriptomic data from nearly 16,000 primary cardiac cells with known specific cell types. This model was then applied to mouse embryonic fibroblasts (MEFs) using a library of 48 cardiac factors as well as 10 epicardial-related factors curated from the literature and bulk-RNA-seq data for perturbations. The results of this approach identified a combination of three transcription factors —Atf3, Gata6, and Hand2— as the primary cocktail to efficiently reprogram MEFs into cells that resemble epicardial cells transcriptionally, molecularly, functionally, and morphologically [97]. While the end goal of this transition was targeted toward epicardial-like cells and not cardiomyocytes, Reprogam-seq could similarly be applied to direct cardiac reprogramming to further validate the current cocktails and potentially identify new factors to be validated experimentally. 

A final example of a promising machine learning platform to model reprogramming is DeepNEU —a model that simulates the reprogramming of artificially induced pluripotent stem cells (aiPSCs) into other targeted cell types using defined sets of reprogramming factors from the literature [98]. Each factor combination used for each simulation can be evaluated for its potential efficacy in converting aiPSCs into the target cell type based on the number of iterations the model must undergo before the reprogrammed cells achieve a gene expression profile specific to the target cell type. This was applied to simulate the conversion of aiPSCs into cardiomyocytes (aiCMCs) using Activin A and BMP4 as the leading reprogramming factors, with the model converging after 15 iterations to a cardiomyocyte-marker-specific expression profile [98]. 

A main caveat of computational reprogramming models is that the reference datasets used to train the models are crucial for the integrity of the resulting predictions [56]. Nonetheless, as this model and others show, machine learning is becoming a crucial tool in streamlining the search for factors that can optimize direct cardiac reprogramming and in artificially modeling the effects of novel cocktails as an efficient antecedent for more costly in vitro and in vivo studies. 

## 6. Closing Perspectives

Direct cardiac reprogramming has great potential to affect countless lives by creating a life-saving alternative trajectory for cells in the injured myocardium. Significant progress in the field has been made in just the past decade, leading to an improved understanding of the molecular mechanisms at play within this process and increased efficiency of reprogramming. As discussed in this review, the integration of bioinformatics has been integral to this process and has bridged the gap between basic reprogramming studies and future clinical applications. The vast amount of data amassed from being able to computationally process transcriptomic, epigenomic, and proteomic data has made the inner workings of direct reprogramming less obscure. The contributions of each factor in the MGT cocktail are far better understood than when they were first introduced in 2010, and the complex temporal trajectory of the reprogrammed cells is more clearly defined. We are now more aware of the strengths of direct cardiac reprogramming, as well as the areas that require more focused attention for improvement –such as in mitigating fibroblast marker gene expression in the final iCM state. Furthermore, machine learning has opened the door to streamlining the process of optimizing direct cardiac reprogramming in ways that would otherwise not be as efficient using purely experimental approaches. 

There is still a long way between the current state of direct cardiac reprogramming and its future clinical application. However, if the last decade has given us any reassurance, it is that tools of computational biology have previously served and will continue to play a valuable role in advancing the field.

## Data Availability

No new data were created or analyzed in this study. Data sharing is not applicable to this article.

## References

[1] Centers for Disease Control and Prevention (CDC) (1999). Decline in deaths from heart disease and stroke—United States, 1900–1999. MMWR Morb. Mortal. Wkly. Rep..

[2] Di Cesare M., Perel P., Taylor S., Kabudula C., Bixby H., Gaziano T.A., McGhie D.V., Mwangi J., Pervan B., Narula J. (2024). The Heart of the World. Glob. Heart.

[3] Tsao C.W., Aday A.W., Almarzooq Z.I., Anderson C.A.M., Arora P., Avery C.L., Baker-Smith C.M., Beaton A.Z., Boehme A.K., Buxton A.E. (2023). Heart Disease and Stroke Statistics—2023 Update: A Report From the American Heart Association. Circulation.

[4] Nowbar A.N., Gitto M., Howard J.P., Francis D.P., Al-Lamee R. (2019). Mortality From Ischemic Heart Disease. Circulation: Cardiovasc. Qual. Outcomes.

[5] Laflamme M.A., Murry C.E. (2005). Regenerating the heart. Nat. Biotechnol..

[6] Bergmann O., Zdunek S., Felker A., Salehpour M., Alkass K., Bernard S., Sjostrom S.L., Szewczykowska M., Jackowska T., dos Remedios C. (2015). Dynamics of Cell Generation and Turnover in the Human Heart. Cell.

[7] Bergmann O., Bhardwaj R.D., Bernard S., Zdunek S., Barnabé-Heider F., Walsh S., Zupicich J., Alkass K., Buchholz B.A., Druid H. (2009). Evidence for cardiomyocyte renewal in humans. Science.

[8] Ieda M., Tsuchihashi T., Ivey K.N., Ross R.S., Hong T.-T., Shaw R.M., Srivastava D. (2009). Cardiac Fibroblasts Regulate Myocardial Proliferation through β1 Integrin Signaling. Dev. Cell.

[9] (2022). NIH NHLBI Heart Failure—Treatment. https://www.nhlbi.nih.gov/health/heart-failure/treatment.

[10] (2023). NIH NHLBI Coronary Heart Disease—Treatment. https://www.nhlbi.nih.gov/health/coronary-heart-disease/treatment.

[11] Cameli M., Pastore M.C., Campora A., Lisi M., Mandoli G.E. (2022). Donor shortage in heart transplantation: How can we overcome this challenge?. Front. Cardiovasc. Med..

[12] Takahashi K., Yamanaka S. (2006). Induction of pluripotent stem cells from mouse embryonic and adult fibroblast cultures by defined factors. Cell.

[13] Zeineddine D., Papadimou E., Mery A., Ménard C., Pucéat M. (2005). Cardiac commitment of embryonic stem cells for myocardial repair. Methods Mol. Med..

[14] Laflamme M.A., Chen K.Y., Naumova A.V., Muskheli V., Fugate J.A., Dupras S.K., Reinecke H., Xu C., Hassanipour M., Police S. (2007). Cardiomyocytes derived from human embryonic stem cells in pro-survival factors enhance function of infarcted rat hearts. Nat. Biotechnol..

[15] Gao L., Gregorich Z.R., Zhu W., Mattapally S., Oduk Y., Lou X., Kannappan R., Borovjagin A.V., Walcott G.P., Pollard A.E. (2018). Large Cardiac Muscle Patches Engineered From Human Induced-Pluripotent Stem Cell-Derived Cardiac Cells Improve Recovery From Myocardial Infarction in Swine. Circulation.

[16] Hassink R.J., Pasumarthi K.B., Nakajima H., Rubart M., Soonpaa M.H., Brutel de la Rivière A., Doevendans P.A., Field L.J. (2008). Cardiomyocyte cell cycle activation improves cardiac function after myocardial infarction. Cardiovasc. Res..

[17] Pasumarthi K.B.S., Nakajima H., Nakajima H.O., Soonpaa M.H., Field L.J. (2005). Targeted expression of cyclin D2 results in cardiomyocyte DNA synthesis and infarct regression in transgenic mice. Circ. Res..

[18] Woo Y.J., Panlilio C.M., Cheng R.K., Liao G.P., Atluri P., Hsu V.M., Cohen J.E., Chaudhry H.W. (2006). Therapeutic delivery of cyclin A2 induces myocardial regeneration and enhances cardiac function in ischemic heart failure. Circulation.

[19] Cheng R.K., Asai T., Tang H., Dashoush N.H., Kara R.J., Costa K.D., Naka Y., Wu E.X., Wolgemuth D.J., Chaudhry H.W. (2007). Cyclin A2 induces cardiac regeneration after myocardial infarction and prevents heart failure. Circ. Res..

[20] Shapiro S.D., Ranjan A.K., Kawase Y., Cheng R.K., Kara R.J., Bhattacharya R., Guzman-Martinez G., Sanz J., Garcia M.J., Chaudhry H.W. (2014). Cyclin A2 induces cardiac regeneration after myocardial infarction through cytokinesis of adult cardiomyocytes. Sci. Transl. Med..

[21] Nakanishi T., Markwald R.R., Baldwin H.S., Keller B.B., Srivastava D., Yamagishi H. (2016). Etiology and Morphogenesis of Congenital Heart Disease: From Gene Function and Cellular Interaction to Morphology.

[22] Aksoz M., Turan R.D., Albayrak E., Kocabas F. (2018). Emerging Roles of Meis1 in Cardiac Regeneration, Stem Cells and Cancer. Curr. Drug Targets.

[23] Nguyen N.U.N., Canseco D., Xiao F., Nakada Y., Li S., Lam N., Muralidhar S.A., Savla J., Hill J.A., Le V. (2020). A Calcineurin-Hoxb13 Axis Regulates Growth Mode of Mammalian Cardiomyocytes. Nature.

[24] Malek Mohammadi M., Kattih B., Grund A., Froese N., Korf-Klingebiel M., Gigina A., Schrameck U., Rudat C., Liang Q., Kispert A. (2017). The transcription factor GATA4 promotes myocardial regeneration in neonatal mice. EMBO Mol. Med..

[25] Fang Y., Lai K.S., She P., Sun J., Tao W., Zhong T.P. (2020). Tbx20 Induction Promotes Zebrafish Heart Regeneration by Inducing Cardiomyocyte Dedifferentiation and Endocardial Expansion. Front. Cell Dev. Biol..

[26] Huang W., Feng Y., Liang J., Yu H., Wang C., Wang B., Wang M., Jiang L., Meng W., Cai W. (2018). Loss of microRNA-128 promotes cardiomyocyte proliferation and heart regeneration. Nat. Commun..

[27] Gabisonia K., Prosdocimo G., Aquaro G.D., Carlucci L., Zentilin L., Secco I., Ali H., Braga L., Gorgodze N., Bernini F. (2019). MicroRNA therapy stimulates uncontrolled cardiac repair after myocardial infarction in pigs. Nature.

[28] Barroso-delJesus A., Romero-López C., Lucena-Aguilar G., Melen G.J., Sanchez L., Ligero G., Berzal-Herranz A., Menendez P. (2008). Embryonic Stem Cell-Specific miR302-367 Cluster: Human Gene Structure and Functional Characterization of Its Core Promoter. Mol. Cell. Biol..

[29] Chen Y., Lüttmann F.F., Schoger E., Schöler H.R., Zelarayán L.C., Kim K.-P., Haigh J.J., Kim J., Braun T. (2021). Reversible reprogramming of cardiomyocytes to a fetal state drives heart regeneration in mice. Science.

[30] Huang W., Dai B., Wen Z., Millard R.W., Yu X.-Y., Luther K., Xu M., Zhao T.C., Yang H.-T., Qi Z. (2013). Molecular Strategy to Reduce In Vivo Collagen Barrier Promotes Entry of NCX1 Positive Inducible Pluripotent Stem Cells (iPSCNCX1+) into Ischemic (or Injured) Myocardium. PLoS ONE.

[31] Dai B., Huang W., Xu M., Millard R.W., Gao M.H., Hammond H.K., Menick D.R., Ashraf M., Wang Y. (2011). Reduced Collagen Deposition in Infarcted Myocardium Facilitates Induced Pluripotent Stem Cell Engraftment and Angiomyogenesis for Improvement of Left Ventricular Function. J. Am. Coll. Cardiol..

[32] Francis Stuart S.D., De Jesus N.M., Lindsey M.L., Ripplinger C.M. (2016). The crossroads of inflammation, fibrosis, and arrhythmia following myocardial infarction. J. Mol. Cell. Cardiol..

[33] Kirmse R., Otto H., Ludwig T. (2011). Interdependency of cell adhesion, force generation and extracellular proteolysis in matrix remodeling. J. Cell Sci..

[34] Kelaini S., Cochrane A., Margariti A. (2014). Direct reprogramming of adult cells: Avoiding the pluripotent state. Stem Cell. Cloning.

[35] Ieda M., Fu J.-D., Delgado-Olguin P., Vedantham V., Hayashi Y., Bruneau B.G., Srivastava D. (2010). Direct Reprogramming of Fibroblasts into Functional Cardiomyocytes by Defined Factors. Cell.

[36] Qian L., Huang Y., Spencer C.I., Foley A., Vedantham V., Liu L., Conway S.J., Fu J., Srivastava D. (2012). In vivo reprogramming of murine cardiac fibroblasts into induced cardiomyocytes. Nature.

[37] Chen J.X., Krane M., Deutsch M.-A., Wang L., Rav-Acha M., Gregoire S., Engels M.C., Rajarajan K., Karra R., Abel E.D. (2012). Inefficient Reprogramming of Fibroblasts into Cardiomyocytes Using Gata4, Mef2c, and Tbx5. Circ. Res..

[38] Inagawa K., Miyamoto K., Yamakawa H., Muraoka N., Sadahiro T., Umei T., Wada R., Katsumata Y., Kaneda R., Nakade K. (2012). Induction of Cardiomyocyte-Like Cells in Infarct Hearts by Gene Transfer of Gata4, Mef2c, and Tbx5. Circ. Res..

[39] Song K., Nam Y.-J., Luo X., Qi X., Tan W., Huang G.N., Acharya A., Smith C.L., Tallquist M.D., Neilson E.G. (2012). Heart repair by reprogramming non-myocytes with cardiac transcription factors. Nature.

[40] Jayawardena T.M., Egemnazarov B., Finch E.A., Zhang L., Payne J.A., Pandya K., Zhang Z., Rosenberg P., Mirotsou M., Dzau V.J. (2012). MicroRNA-Mediated In Vitro and In Vivo Direct Reprogramming of Cardiac Fibroblasts to Cardiomyocytes. Circ. Res..

[41] Protze S., Khattak S., Poulet C., Lindemann D., Tanaka E.M., Ravens U. (2012). A new approach to transcription factor screening for reprogramming of fibroblasts to cardiomyocyte-like cells. J. Mol. Cell. Cardiol..

[42] Addis R.C., Ifkovits J.L., Pinto F., Kellam L.D., Esteso P., Rentschler S., Christoforou N., Epstein J.A., Gearhart J.D. (2013). Optimization of direct fibroblast reprogramming to cardiomyocytes using calcium activity as a functional measure of success. J. Mol. Cell. Cardiol..

[43] Christoforou N., Chellappan M., Adler A.F., Kirkton R.D., Wu T., Addis R.C., Bursac N., Leong K.W. (2013). Transcription Factors MYOCD, SRF, Mesp1 and SMARCD3 Enhance the Cardio-Inducing Effect of GATA4, TBX5, and MEF2C during Direct Cellular Reprogramming. PLoS ONE.

[44] Nam Y.-J., Song K., Luo X., Daniel E., Lambeth K., West K., Hill J.A., DiMaio J.M., Baker L.A., Bassel-Duby R. (2013). Reprogramming of human fibroblasts toward a cardiac fate. Proc. Natl. Acad. Sci. USA.

[45] Muraoka N., Yamakawa H., Miyamoto K., Sadahiro T., Umei T., Isomi M., Nakashima H., Akiyama M., Wada R., Inagawa K. (2014). MiR-133 promotes cardiac reprogramming by directly repressing Snai1 and silencing fibroblast signatures. EMBO J..

[46] Wang L., Liu Z., Yin C., Zhou Y., Liu J., Qian L. (2015). Improved Generation of Induced Cardiomyocytes Using a Polycistronic Construct Expressing Optimal Ratio of Gata4, Mef2c and Tbx5. J. Vis. Exp..

[47] Zhao H., Zhang Y., Xu X., Sun Q., Yang C., Wang H., Yang J., Yang Y., Yang X., Liu Y. (2021). Sall4 and Myocd Empower Direct Cardiac Reprogramming From Adult Cardiac Fibroblasts After Injury. Front. Cell Dev. Biol..

[48] Tani H., Sadahiro T., Yamada Y., Isomi M., Yamakawa H., Fujita R., Abe Y., Akiyama T., Nakano K., Kuze Y. (2023). Direct Reprogramming Improves Cardiac Function and Reverses Fibrosis in Chronic Myocardial Infarction. Circulation.

[49] Wang H., Keepers B., Qian Y., Xie Y., Colon M., Liu J., Qian L. (2022). Cross-lineage potential of Ascl1 uncovered by comparing diverse reprogramming regulatomes. Cell Stem Cell.

[50] Dal-Pra S., Hodgkinson C.P., Mirotsou M., Kirste I., Dzau V.J. (2017). Demethylation of H3K27 Is Essential for the Induction of Direct Cardiac Reprogramming by miR Combo. Circ. Res..

[51] Dal-Pra S., Hodgkinson C.P., Dzau V.J. (2019). Induced cardiomyocyte maturation: Cardiac transcription factors are necessary but not sufficient. PLoS ONE.

[52] Zhou Y., Liu Z., Welch J.D., Gao X., Wang L., Garbutt T., Keepers B., Ma H., Prins J.F., Shen W. (2019). Single-Cell Transcriptomic Analyses of Cell Fate Transitions during Human Cardiac Reprogramming. Cell Stem Cell.

[53] Tang Y., Aryal S., Geng X., Zhou X., Fast V.G., Zhang J., Lu R., Zhou Y. (2022). TBX20 Improves Contractility and Mitochondrial Function During Direct Human Cardiac Reprogramming. Circulation.

[54] Liu Z., Wang L., Welch J.D., Ma H., Zhou Y., Vaseghi H.R., Yu S., Wall J.B., Alimohamadi S., Zheng M. (2017). Single Cell Transcriptomics Reconstructs Fate Conversion from Fibroblast to Cardiomyocyte. Nature.

[55] Hashimoto H., Wang Z., Garry G.A., Malladi V.S., Botten G.A., Ye W., Zhou H., Osterwalder M., Dickel D.E., Visel A. (2019). Cardiac Reprogramming Factors Synergistically Activate Genome-wide Cardiogenic Stage-Specific Enhancers. Cell Stem cell.

[56] Stone N.R., Gifford C.A., Thomas R., Pratt K.J.B., Samse-Knapp K., Mohamed T.M.A., Radzinsky E.M., Schricker A., Ye L., Yu P. (2019). Context-Specific Transcription Factor Functions Regulate Epigenomic and Transcriptional Dynamics during Cardiac Reprogramming. Cell Stem Cell.

[57] Zhou Y., Wang L., Vaseghi H.R., Liu Z., Lu R., Alimohamadi S., Yin C., Fu J.-D., Wang G.G., Liu J. (2016). Bmi1 Is a Key Epigenetic Barrier to Direct Cardiac Reprogramming. Cell Stem Cell.

[58] Garry G.A., Bezprozvannaya S., Chen K., Zhou H., Hashimoto H., Morales M.G., Liu N., Bassel-Duby R., Olson E.N. (2021). The histone reader PHF7 cooperates with the SWI/SNF complex at cardiac super enhancers to promote direct reprogramming. Nat. Cell Biol..

[59] Sauls K., Greco T.M., Wang L., Zou M., Villasmil M., Qian L., Cristea I.M., Conlon F.L. (2018). Initiating Events in Direct Cardiomyocyte Reprogramming. Cell Rep..

[60] Bernhardt O.M., Selevsek N., Gillet L.C., Rinner O., Picotti P., Aebersold R., Reiter L. (2012). Spectronaut A fast and efficient algorithm for MRM-like processing of data independent acquisition (SWATH-MS) data. F1000Research.

[61] Wang J., Jiang X., Zhao L., Zuo S., Chen X., Zhang L., Lin Z., Zhao X., Qin Y., Zhou X. (2020). Lineage reprogramming of fibroblasts into induced cardiac progenitor cells by CRISPR/Cas9-based transcriptional activators. Acta Pharm. Sin. B.

[62] Jiang L., Liang J., Huang W., Ma J., Park K.H., Wu Z., Chen P., Zhu H., Ma J.-J., Cai W. (2022). CRISPR activation of endogenous genes reprograms fibroblasts into cardiovascular progenitor cells for myocardial infarction therapy. Mol. Ther..

[63] Yu J.S.L., Palano G., Lim C., Moggio A., Drowley L., Plowright A.T., Bohlooly-Y M., Rosen B.S., Hansson E.M., Wang Q.-D. (2019). CRISPR-Knockout Screen Identifies Dmap1 as a Regulator of Chemically Induced Reprogramming and Differentiation of Cardiac Progenitors. Stem Cells.

[64] Cahan P., Li H., Morris S.A., da Rocha E.L., Daley G.Q., Collins J.J. (2014). CellNet: Network Biology Applied to Stem Cell Engineering. Cell.

[65] Kong W., Fu Y.C., Holloway E.M., Garipler G., Yang X., Mazzoni E.O., Morris S.A. (2022). Capybara: A computational tool to measure cell identity and fate transitions. Cell Stem Cell.

[66] Rackham O.J.L., Firas J., Fang H., Oates M.E., Holmes M.L., Knaupp A.S., Suzuki H., Nefzger C.M., Daub C.O., FANTOM Consortium (2016). A predictive computational framework for direct reprogramming between human cell types. Nat. Genet..

[67] Romero-Tejeda M., Fonoudi H., Weddle C.J., DeKeyser J.-M., Lenny B., Fetterman K.A., Magdy T., Sapkota Y., Epting C.L., Burridge P.W. (2023). A novel transcription factor combination for direct reprogramming to a spontaneously contracting human cardiomyocyte-like state. J. Mol. Cell. Cardiol..

[68] Wu X., Yang B., Udo-Inyang I., Ji S., Ozog D., Zhou L., Mi Q.-S. (2018). Research Techniques Made Simple: Single-Cell RNA Sequencing and its Applications in Dermatology. J. Invest. Dermatol..

[69] Welch J.D., Hartemink A.J., Prins J.F. (2016). SLICER: Inferring branched, nonlinear cellular trajectories from single cell RNA-seq data. Genome Biol..

[70] Abe Y., Tani H., Sadahiro T., Yamada Y., Akiyama T., Nakano K., Honda S., Ko S., Anzai A., Ieda M. (2024). Cardiac reprogramming reduces inflammatory macrophages and improves cardiac function in chronic myocardial infarction. Biochem. Biophys. Res. Commun..

[71] Buenrostro J.D., Giresi P.G., Zaba L.C., Chang H.Y., Greenleaf W.J. (2013). Transposition of native chromatin for fast and sensitive epigenomic profiling of open chromatin, DNA-binding proteins and nucleosome position. Nat. Methods.

[72] Robertson G., Hirst M., Bainbridge M., Bilenky M., Zhao Y., Zeng T., Euskirchen G., Bernier B., Varhol R., Delaney A. (2007). Genome-wide profiles of STAT1 DNA association using chromatin immunoprecipitation and massively parallel sequencing. Nat. Methods.

[73] Liu Z., Chen O., Zheng M., Wang L., Zhou Y., Yin C., Liu J., Qian L. (2016). Re-patterning of H3K27me3, H3K4me3 and DNA methylation during fibroblast conversion into induced cardiomyocytes. Stem Cell Res..

[74] Wang H., Yang Y., Qian Y., Liu J., Qian L. (2022). Delineating chromatin accessibility re-patterning at single cell level during early stage of direct cardiac reprogramming. J. Mol. Cell. Cardiol..

[75] Mi H., Muruganujan A., Huang X., Ebert D., Mills C., Guo X., Thomas P.D. (2019). Protocol Update for Large-scale genome and gene function analysis with PANTHER Classification System (v.14.0). Nat. Protoc..

[76] Mathison M., Singh V.P., Sanagasetti D., Yang L., Pinnamaneni J.P., Yang J., Rosengart T.K. (2017). Cardiac reprogramming factor Gata4 reduces postinfarct cardiac fibrosis through direct repression of the profibrotic mediator snail. J. Thorac. Cardiovasc. Surg..

[77] Wang H., Yang J., Cai Y., Zhao Y. (2024). Macrophages suppress cardiac reprogramming of fibroblasts in vivo via IFN-mediated intercellular self-stimulating circuit. Protein Cell.

[78] Orsburn B.C. (2021). Proteome Discoverer—A Community Enhanced Data Processing Suite for Protein Informatics. Proteomes.

[79] Bouyssié D., Gonzalez de Peredo A., Mouton E., Albigot R., Roussel L., Ortega N., Cayrol C., Burlet-Schiltz O., Girard J.-P., Monsarrat B. (2007). Mascot file parsing and quantification (MFPaQ), a new software to parse, validate, and quantify proteomics data generated by ICAT and SILAC mass spectrometric analyses: Application to the proteomics study of membrane proteins from primary human endothelial cells. Mol. Cell. Proteom..

[80] Zhang J., Xin L., Shan B., Chen W., Xie M., Yuen D., Zhang W., Zhang Z., Lajoie G.A., Ma B. (2012). PEAKS DB: De novo sequencing assisted database search for sensitive and accurate peptide identification. Mol. Cell. Proteom..

[81] Bachamanda Somesh D., Klose K., Maring J.A., Kunkel D., Jürchott K., Protze S.I., Klein O., Nebrich G., Becker M., Krüger U. (2023). Cardiomyocyte precursors generated by direct reprogramming and molecular beacon selection attenuate ventricular remodeling after experimental myocardial infarction. Stem Cell Res. Ther..

[82] Jinek M., Chylinski K., Fonfara I., Hauer M., Doudna J.A., Charpentier E. (2012). A Programmable Dual-RNA–Guided DNA Endonuclease in Adaptive Bacterial Immunity. Science.

[83] Ståhl P.L., Salmén F., Vickovic S., Lundmark A., Navarro J.F., Magnusson J., Giacomello S., Asp M., Westholm J.O., Huss M. (2016). Visualization and analysis of gene expression in tissue sections by spatial transcriptomics. Science.

[84] Asp M., Giacomello S., Larsson L., Wu C., Fürth D., Qian X., Wärdell E., Custodio J., Reimegård J., Salmén F. (2019). A Spatiotemporal Organ-Wide Gene Expression and Cell Atlas of the Developing Human Heart. Cell.

[85] Farah E.N., Hu R.K., Kern C., Zhang Q., Lu T.-Y., Ma Q., Tran S., Zhang B., Carlin D., Monell A. (2024). Spatially organized cellular communities form the developing human heart. Nature.

[86] Kanemaru K., Cranley J., Muraro D., Miranda A.M.A., Ho S.Y., Wilbrey-Clark A., Patrick Pett J., Polanski K., Richardson L., Litvinukova M. (2023). Spatially resolved multiomics of human cardiac niches. Nature.

[87] Kuppe C., Ramirez Flores R.O., Li Z., Hayat S., Levinson R.T., Liao X., Hannani M.T., Tanevski J., Wünnemann F., Nagai J.S. (2022). Spatial multi-omic map of human myocardial infarction. Nature.

[88] Liu X., Yin K., Chen L., Chen W., Li W., Zhang T., Sun Y., Yuan M., Wang H., Song Y. (2023). Lineage-specific regulatory changes in hypertrophic cardiomyopathy unraveled by single-nucleus RNA-seq and spatial transcriptomics. Cell Discov..

[89] Misra A., Baker C.D., Pritchett E.M., Burgos Villar K.N., Ashton J.M., Small E.M. (2021). Characterizing Neonatal Heart Maturation, Regeneration, and Scar Resolution Using Spatial Transcriptomics. J. Cardiovasc. Dev. Dis..

[90] Williams C.G., Lee H.J., Asatsuma T., Vento-Tormo R., Haque A. (2022). An introduction to spatial transcriptomics for biomedical research. Genome Med..

[91] Janesick A., Shelansky R., Gottscho A.D., Wagner F., Williams S.R., Rouault M., Beliakoff G., Morrison C.A., Oliveira M.F., Sicherman J.T. (2023). High resolution mapping of the tumor microenvironment using integrated single-cell, spatial and in situ analysis. Nat. Commun..

[92] Abdelaal T., Michielsen L., Cats D., Hoogduin D., Mei H., Reinders M.J.T., Mahfouz A. (2019). A comparison of automatic cell identification methods for single-cell RNA sequencing data. Genome Biol..

[93] Alquicira-Hernandez J., Sathe A., Ji H.P., Nguyen Q., Powell J.E. (2019). scPred: Accurate supervised method for cell-type classification from single-cell RNA-seq data. Genome Biol..

[94] Tan Y., Cahan P. (2019). SingleCellNet: A Computational Tool to Classify Single Cell RNA-Seq Data Across Platforms and Across Species. Cell Syst..

[95] Pliner H.A., Shendure J., Trapnell C. (2019). Supervised classification enables rapid annotation of cell atlases. Nat. Methods.

[96] Tran A., Yang P., Yang J.Y.H., Ormerod J.T. (2022). scREMOTE: Using multimodal single cell data to predict regulatory gene relationships and to build a computational cell reprogramming model. NAR Genom. Bioinform..

[97] Duan J., Li B., Bhakta M., Xie S., Zhou P., Munshi N.V., Hon G.C. (2019). Rational Reprogramming of Cellular States by Combinatorial Perturbation. Cell Rep..

[98] Danter W.R. (2019). DeepNEU: Cellular reprogramming comes of age—A machine learning platform with application to rare diseases research. Orphanet J. Rare Dis..

